# Multivariate Chemical Image Fusion of Vibrational Spectroscopic Imaging Modalities

**DOI:** 10.3390/molecules21070870

**Published:** 2016-07-02

**Authors:** Aoife A. Gowen, Ronan M. Dorrepaal

**Affiliations:** UCD School of Biosystems and Food Engineering, College of Engineering and Architecture, University College Dublin, Belfield, Dublin 4, Ireland; ronan.dorrepaal@ucdconnect.ie

**Keywords:** hyperspectral, data, fusion, vibration, spectroscopic, imaging, multivariate, chemometrics

## Abstract

Chemical image fusion refers to the combination of chemical images from different modalities for improved characterisation of a sample. Challenges associated with existing approaches include: difficulties with imaging the same sample area or having identical pixels across microscopic modalities, lack of prior knowledge of sample composition and lack of knowledge regarding correlation between modalities for a given sample. In addition, the multivariate structure of chemical images is often overlooked when fusion is carried out. We address these challenges by proposing a framework for multivariate chemical image fusion of vibrational spectroscopic imaging modalities, demonstrating the approach for image registration, fusion and resolution enhancement of chemical images obtained with IR and Raman microscopy.

## 1. Introduction

Vibrational spectroscopic techniques have become standard in a wide variety of scientific fields, some of the most common being near-infrared (NIR), mid-infrared (MIR) and Raman spectrometry. These techniques have been used to great effect, however traditionally researchers relied on average spectroscopic data from a single sample point or, often in the case of materials, from the average spectrum of a material after pulverisation. Chemical imaging takes spectroscopy beyond this limitation. Where standard spectroscopy acquires chemical information from a single region, chemical imaging collects chemical information over many spatial regions. These regions can be used to form a high resolution matrix image over an area, where each element is an image pixel comprising a spectrum. Chemical variation across the surface of a material can therefore be assessed. Such information is used to provide an insight into the properties of foods [1,2], survey the geological and vegetal make up of landscapes [3] or improve synthetic processes through a better understanding of their resulting products [4].

While chemical imaging provides a marked advantage over standard spectroscopy, limitations associated with the various spectroscopy modalities also apply to corresponding chemical imaging techniques. For example, certain molecular features, such as asymmetric molecular vibrations are invisible or near invisible to Raman spectroscopy. Equivalently, symmetric molecular vibrations are invisible or near invisible to MIR spectroscopy. Additionally, the spatial resolution achievable by all spectral modalities is restricted by the diffraction limit which differs by modality.

One method adopted for the purposes of overcoming these limitations is the fusion of different techniques. Fusion can refer to the combination of two or more chemical image cubes to arrive at a greater insight than the sum of their parts. Alternatively, fusion can refer to the combination of a high spatial resolution image comprising little or no spectral information with an image with relatively low spatial resolution but higher spectral resolution [3]. Where a common area is imaged by different imaging techniques, one fusion approach has been the overlaying of two or more images at a specific absorption band such that the physical detail of the high spatial resolution image is visible with a colour scheme denoting different chemical regions elucidated from the imaging technique with greater spectral information [5,6]. The above examples are often called multi-model fusion techniques, where images from different modalities are fused, however mono-modal fusion is also possible. For example, mono-modal fusion, where images are taken using the same modality, but at different spatial resolutions, or at different time points, can also be performed.

Clarke et al. [5] were amongst the first to consider the combination of chemical imaging techniques, opting to experiment with FT-NIR imaging and complementary Raman point mapping. The study was performed in the context of visualising pharmaceutical formulations. The approach involved the imaging of a specific sample area using both techniques. The sample area was delineated with reference markers and with careful consideration of these it was possible to obtain Raman and NIR images of the same sample area with the same number of spatial pixels in each cube. Chemical image fusion, a term coined by the authors, was performed by overlaying several NIR and Raman images at wavenumbers specific to different components within the pharmaceutical blend. This study was very effective as the techniques proved to be complementary and each technique aided in removing ambiguities which would have been present if only one technique where used. 

Multi-way chemometric approaches have also been explored for coupling chemical imaging data from different microspectroscopic modalities. For example, co-inertia (also known as Tucker) analysis is a multiway method that can be used to relate two data matrices with rows representing the same image pixels. In one paper [7], Allouche et al. used multiple co-inertia analysis to combine three hyperspectral images of maize sections with different spatial resolutions, obtained using IR, fluorescence and Raman microspectroscopy. Prior to co-inertia analysis, the three hyperspectral images were co-registered to brightfield images and differences in spatial resolution were removed by pixel averaging. The initial resolution of the Raman and fluorescence images was recovered by projecting the original images along the block or global loadings obtained. In a related paper [8], Allouche et al coupled fluorescence and IR images of a maize cross section using an extended co-inertia approach. Each 1 × 1 pixel in the IR data was related to a 7 × 7 pixel in the higher resolution fluorescence data by registration to brightfield images. The co-inertia approach was extended to simultaneously analyse a two and a three way data table, where the two way table comprised the unfolded IR image and the three way table comprised the unfolded fluorescence image (the third way representing the 49 pixels of fluorescence data for each IR pixel). The authors demonstrated that the extended analysis enabled preservation of the higher resolution of the fluorescence data while not affecting the spectral interpretation of co-inertia loadings.

In a more recent study, Ewing et al. [4] obtained ATR-IR and Raman chemical image cubes of pharmaceutical tablet samples of different sizes. The tablets comprised an API trapped within a polymer matrix which swelled when exposed to an appropriate liquid, exposing the API particles to the liquid. The team argued that while the solubility was known for the API, if disproportionation (i.e., the API ion reverting to the free acid form) occurred, the dissolution rate would change markedly as the free acid is less soluble in a polar solvent. The experiments were conducted in a flow cell and various pH solutions were used. The two modalities were used to image both tablets and investigate for spatial variation, particularly searching for the presence of the API ion and free acid forms. Though the imaging modalities were performed on different samples and no multivariate data fusion was conducted, the results complemented one another and confirmed that different pH levels, different amounts of disproportionation occur.

Data fusion is also useful in the field of remote sensing. For example, Licciardi et al. [3] described the combination of hyperspectral (such as a Compact High Resolution Imaging Spectrometer (CHRIS) sensor acquisition of 63 spectral bands in range of 400–1050 nm using) and panchromatic images (such as a Quickbird-PAN acquisition of a black and white image generated over the wavelength range of 405–1050 nm). The approach incorporates dimensionality reduction and indusion (induction and fusion). The team’s main aim was the fusion of both modalities to arrive at an image with the high spatial resolution of panchromatic imaging and the chemical information of chemical imaging. The proposed fusion approach involved performing non-linear principle component analysis (NLPCA) on the chemical image cube for dimensionality reduction purposes, followed by down-scaling of the panchromatic image using a filter to fit the size of the NLPCs. Histogram-matching was then performed between the NLPCs and the down-sized panchromatic image. The NLPCs were up-scaled as was the histogram-matched panchromatic image. Histogram-matching was then performed between the up-scaled NLPCs and the original panchromatic image. The difference between the histogram-matched up-scaled panchromatic image and the histogram-matched original panchromatic image was found and this difference was added to the up-scaled NLPCs. The original spectral bands were reconstructed through decoding. Visual quality assessments of the resultant image were then conducted. The spectra were also altered in the indusion process and the root mean square error (RMSE) was calculated between the produced spectra and the original spectra and while some issues were reported for features that were visible in the pan chromatic image but not in the chemical image cube, the results appear to be quite favourable.

Fusion has also been used in non-vibrational spectroscopic imaging modalities for material characterisation. For example, Artyushkova et al. [6] describes a method of fusion of X-ray photoelectron spectroscopy (XPS) and atomic force microscopy (AFM) images of polymer blends. AFM has nanometer spatial resolution while XPS provides spectral information but low spatial information. The fusion technique requires the de-resolution of the AFM image such that the resulting image has the same spatial resolution as the XPS. The images were registered to each other using automatic image registration (AIR). This translates, rotates and scales the pixels appropriately. The mapping can then be checked and confirmed. When the necessary translation, rotation and scaling is known, the original AFM image can then be used and the chemical detail of the XPS image overlaid and presented as colour changes. This results in an image with the high spatial resolution of AFM and the chemical information of XPS. Van de Plas et al. [9] describes the use of data fusion through the combination of imaging mass spectrometry (IMS) and high resolution light microscopy. The imaging techniques were combined to take advantage of the high spatial resolution of light microscopy and the chemical information of lower resolution IMS. The team aimed to combine ion intensity readings with photon based variables by modelling the distribution of each throughout the area of the images. This required the use of partial least squares regression (PLSR), which predicted ion concentrations at different regions of brain tissue. It is reported that the microscopy image (the spatially distributed photon variables) could predict ion distribution with a reasonable degree of accuracy for several ion mass/charge ratios. In addition, the team reported that looking at modalities in tandem was useful in confirming signals that would otherwise have been considered instrument noise. 

Challenges associated with data fusion methods should also be noted. A first such challenge may be any destructive effect that one imaging technique has on a sample, particularly as this could affect subsequent readings on other modalities. For example, mass spectrometry chemical imaging necessarily removes small amounts of sample in order to conduct analysis, which will affect a sample to some extent, however small. Further, Raman spectroscopy, while not generally considered a destructive technique, does require the focusing of a laser on a sample and can therefore lead to small amounts of sample evaporation/sublimation or provide the activation energy required for a new reaction to take place, including but not limited to sample combustion in oxygen. One solution may be to order imaging modalities with increasing destructiveness so as to reduce sampling artefacts. In addition, when an experiment involves imaging over a time series for a dynamic sample, the experimenter must decide on a compromise, choosing to image of same the sample at different times, or image different but similar samples at the same time. Both approaches unfortunately reduce experimental certainty. Further challenges include: potential difficulties with imaging the same sample area across microscopic modalities, lack of prior knowledge of sample composition and or availability of pure component spectra.

Our literature survey indicates that the multivariate nature of chemical image data is often neglected in data fusion (e.g., [4,5]). Multivariate chemical fusion techniques differ from standard fusion in that the imaging techniques used are not considered in mathematic isolation. Multivariate chemical fusion techniques therefore have at least two very significant advantages over standard fusion techniques. The first is the reduction of experimenter bias in the designation of signal correlations between features of different imaging techniques. This is because a sole reliance on visual interpretation is no longer required. Further, correlations between imaging techniques which are not intuitively obvious to an experimenter visually may be found with multivariate chemical fusion. Therefore, multivariate chemical fusion approaches increase certainty by serving to reduce both false positives and false negatives in relation to inter-modality correlations.

In this paper we provide a framework for multivariate chemical image (CI) fusion to enable: cross modality image registration; improved classification performance; investigation of cross modality correlations; prediction of one modality from another and resolution enhancement. 

## 2. Multivariate CI Registration

**Figure 1:**
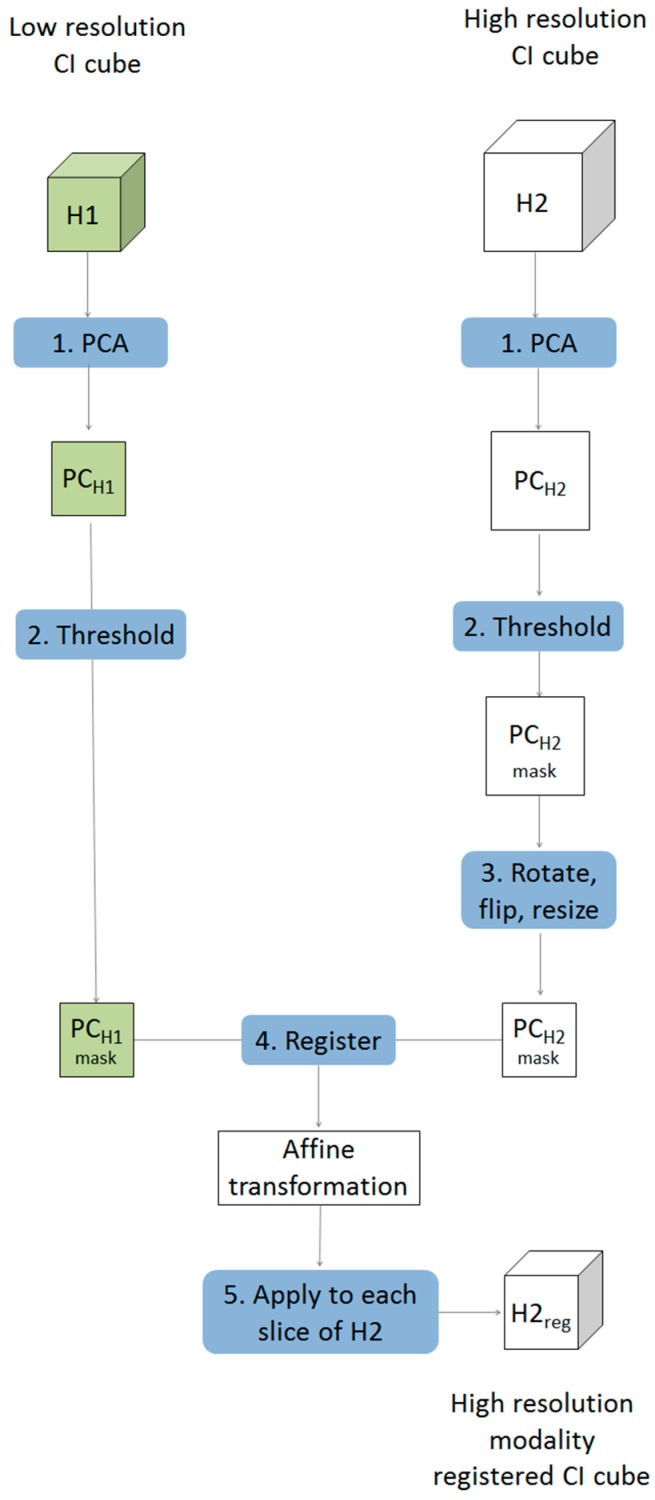
Schematic showing framework for multivariate chemical image cube registration. Starting with a low resolution modality (H1) and a higher resolution modality (H2), the higher resolution image is rotated, resampled and transformed resulting in order to register it to match the spatial resolution of the lower resolution image.

Multivariate image registration was achieved in five steps as described below and shown schematically in Figure 1.

1. Starting with two chemical image cubes, one obtained using a lower resolution modality (H1) and the other obtained using a higher resolution modality (H2), principal component analysis (PCA) was applied to each chemical image cube individually. The resultant PC score images were visually examined and compared between modes. One PC score image, corresponding to common salient features of the sample, was selected for each modality (PC_H1_ & PC_H2_).
2. The PC score images selected in step 1 were thresholded, based on their histograms, to create a binary mask exposing the common salient features.
3. The higher resolution PC mask image was rotated and flipped to match the orientation of the lower resolution one. This was followed by down-sampling of the higher resolution image to the approximate size of the lower resolution image, resulting in a PC score mask image for the 2nd modality (PC_H2r_) with similar orientation and size to that of the 1st modality (PC_H1_). Images were down-sampled using the “imresize” function of the Matlab image processing toolbox [10]. This function works by resizing a two dimensional image to a target size using bicubic interpolation.
4. An affine transformation to register the down-sampled image to the lower resolution image was optimised using a regular step gradient descent method. Image registration parameters were obtained from the binary masks created in step 3 and optimized using the “imregtform” function of the Matlab image processing toolbox [10]. This function takes two input images of the same size: one is regarded as fixed, this is the reference image, and the other is regarded as moving, this is the image that will be transformed to match the spatial arrangement of the fixed image. The transformation is defined by optimising a criterion, in this case study the mean square difference between the fixed and transformed image was minimised. The output of this function is an affine transformation which can then be applied to individual slices of the chemical image that requires registration.
5. Each slice of the higher resolution chemical image cube (H2) was then rotated, flipped, resized (according to the procedure defined in step 3) and the affine transformation optimised in step 4 was applied, resulting in a new registered chemical image cube where each pixel matches that of the lower resolution one.

## 3. Multivariate CI Fusion

**Figure 2:**
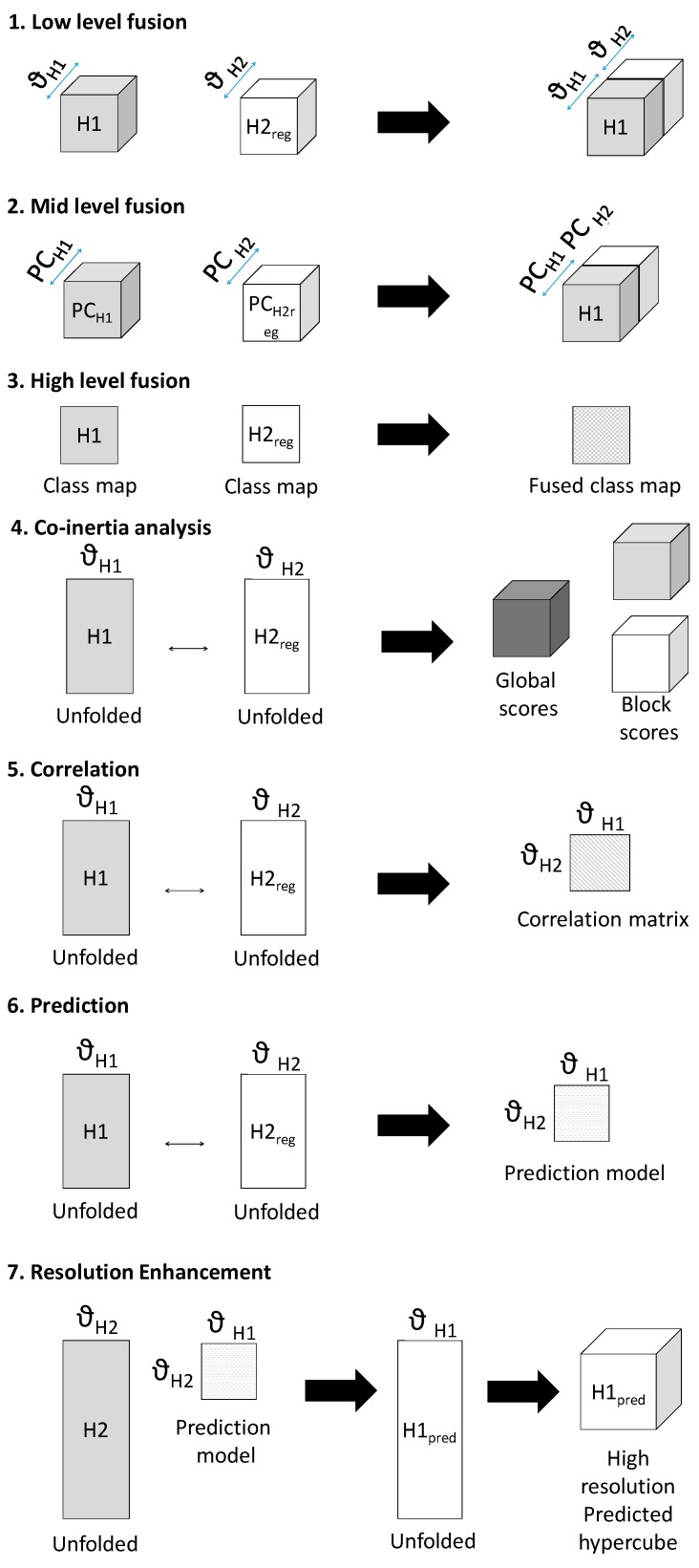
Schematic showing framework of multivariate chemical image cube fusion methods investigated. Starting with a low resolution modality chemical image cube (H1) and a registered higher resolution modality chemical image cube (H2_reg_), the different levels of image fusion (low, medium and high) are represented. Co-inertia analysis, correlation and prediction for resolution enhacement are also shown schematically.

Seven methods of data fusion are presented here, as described below and shown schematically in Figure 2:

1. Low-level fusion: spatially registered image cubes were concatenated to make a fused chemical image cube. Multivariate analysis (i.e., pixel classification using partial least squares discriminant analysis (PLS-DA—further details are given in the data analysis section) was applied to this chemical image cube.
2. Mid-level fusion: outputs from data reduction (i.e., PCA) were fused, followed by pixel classification.
3. High-level fusion: outputs from classification applied to each cube separately were fused.
4. Co-inertia analysis: unfolded, registered cubes were subjected to co-inertia analysis, as described in [7]. This method generates global co-inertia scores and loadings from concatenated unfolded registered chemical images, sequentially deflated by the contribution of each chemical image (or “block”) to the global co-inertia scores and loadings. This enables analysis of the contribution of each data block to the overall covariance in the global dataset described by each co-inertia component and examination of the correlation between each block and global component. Further details on the metrics extracted from the co-inertia analysis are given in the data analysis section.
5. Correlation analysis: the Pearson correlation coefficient [11] between spectra from the spatially registered data was calculated to reveal spectral correlations between modalities.
6. Prediction: multivariate regression models were built to predict spectra from one modality from the other. The registered data was divided into training, calibration and test data and PLSR models were developed to predict intensity at a given spectral variable. Further details on the PLSR models are given in the data analysis section.
7. Resolution enhancement: Using the predictive models developed in method 6, IR chemical image cubes were predicted from high resolution Raman chemical image cubes. The predicted images were evaluated by comparison with the actual high resolution IR image.

## 4. Results & Discussion

### 4.1. Multivariate Image Registration

Prior to multivariate chemical image fusion, it is necessary to ensure that the pixels represented by each technique cover the same spatial area of the sample. This can be achieved by multivariate image registration, as described in Section 2. The results for multivariate image registration, following the five steps shown in Figure 1, are described below.

#### 4.1.1. Selection of Target Images for Image Registration

**Figure 3:**
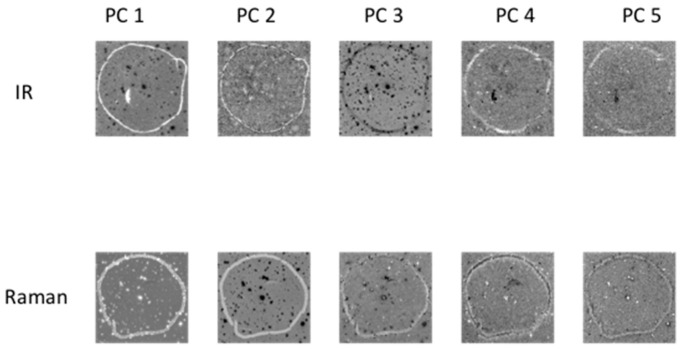
PCA applied to chemical image cubes from different modalities (image set 1): First five IR and Raman PC score images are shown. The main salient feature used for registration was the loop feature, which is seen in IR PC 1 and in Raman PC 2 as a bright loop. All images were scaled to the range mean ± 4 standard deviations.

Principal component analysis (PCA) was applied to each chemical image cube individually. PC component images were inspected and compared between modalities as shown in Figure 3 (for image set 1) and Figure S1 (for image set 2). For image set 1, the main salient feature used for registration was the outline feature, which is seen most prominently in IR PC 1 and in Raman PC 2 as a bright loop. Therefore IR PC 1 and Raman PC 2 score images were selected as inputs for image registration. PC component images for image set 2 are shown in Figure S1; in this dataset, PC 1 images from both modalities were chosen for registration.

#### 4.1.2. Conversion of Target Images into Mask Images

**Figure 4:**
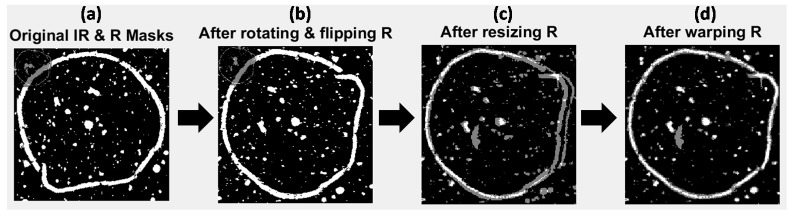
Multivariate image registration for image set 1. Gray pixels show mis-matching pixels, white pixels show matching pixels. (**a**) Original IR and Raman mask images overlaid; (**b**) IR and Raman mask images overlaid after rotating and flipping Raman mask; (**c**) IR and Raman mask images overlaid after rotating, flipping and down-sampling the Raman mask; (**d**) IR and Raman mask images overlaid after rotating, flipping, down-sampling and applying affine transformation to the Raman mask.

The selected PC score images were converted into binary masks by histogram based thresholding, resulting in two mask images, as overlaid in Figure 4a and Figure S2a for image sets 1 and 2 respectively. Overlaying the two images shows the difference in size, resolution and orientation of the images due to inherent image acquisition differences between the two modalities. In the case of image set 3 (the set USAF resolution target images) grayscale rather than thresholded PC images were used as targets, since thresholding introduced errors into the mask images (see Figure S8a).

#### 4.1.3. Rotation of Mask Images

Image set 1: The higher resolution Raman mask image was rotated and flipped to match the orientation of the lower resolution IR mask image. This was followed by down-sampling of the Raman image to the size of the lower resolution IR mask (from 787 × 774 pixels to 196 × 190 pixels). Down-sampling of the higher resolution image (rather than up-sampling of the lower resolution image) was carried out to better represent the same areas imaged by both techniques. The effect of these processes is shown in Figure 4b,c, where the IR and transformed (rotated, flipped and resized) Raman mask images are overlaid. The masks now start to look more similar than before, however the salient loop feature does not match in both images, since the pixel size of the lower resolution image was not a whole number multiple of that of the higher resolution one (25 μm versus 6.2 μm). Image sets 2 and 3: Similar procedures for image sets 2 and 3 are shown in the Supplementary Material (Figures S2 and S8). Image set 2 did not require rotating or flipping since the NIR and Vis-NIR reflectance images were of the same orientation.

#### 4.1.4. Registration of Target Images

Image set 1: After rotating, flipping and down-sampling, the Raman mask was registered to the IR mask by optimisation of an affine transformation to map the pixel positions in the Raman mask to those in the IR mask. The resultant transformed mask is overlaid with the IR mask in Figure 4d. It can be seen that the outline feature now matches up well (white pixels in Figure 4d). However, some pixels present in the IR mask that are not found in the transformed Raman mask (gray pixels in Figure 4c) are evident. These non-matching pixels correspond to features that are not common to IR PC 1 and Raman PC 2 score images. Image sets 2 and 3: The results of applying similar procedures to image sets 2 and 3 are shown in the supplementary material (Figures S2 and S8).

#### 4.1.5. Multivariate Chemical Image Registration

Each slice of the original high resolution chemical image was subjected to the rotation, flipping, resampling and transformation steps defined by the analysis of the mask images, resulting in a new registered chemical image cube where each pixel position matched that of the lower resolution chemical image cube.

### 4.2. Multivariate Data Fusion

#### 4.2.1. Multivariate Data Fusion for Pixel Classification

**Figure 5:**
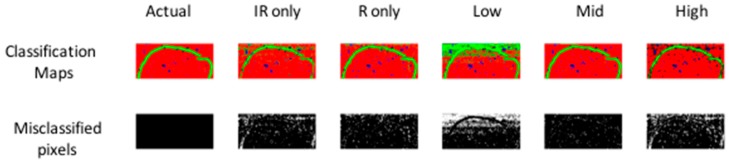
PLS-DA pixel classification for models developed on separate IR, separate Raman, low-, mid- and high-level fused IR-Raman data for image set 1. Actual target classification map is shown in leftmost upper panel. Classification maps for Class 1–3 (red = Class 1, green = Class 2, blue = Class 3) resulting from PLS-DA modelling shown in upper panels. Misclassified pixels for each model shown as white pixels in lower panel. Further model details are shown in Table 1.

After multivariate registration of chemical images, three levels of data fusion (low, mid and high, see also Figure 2 and Section 3), were investigated, as described below. The levels of fusion were compared in terms of the ability of the data set to discriminate between pixel classes in the dataset. A calibration set of spectra for PLS-DA model building was obtained from half of each image cube and the developed calibration model was applied to the remainder, resulting in class prediction maps, as shown in Figure 5 (for image set 1) and Figure S3 (for image set 2). For comparison, the same method was separately applied to individual chemical image cubes. Classification model metrics (number of latent variables and % correct classification) are shown in Table 1 (for image set 1) and Table S1 (for image set 2).

#### 4.2.2. Low-Level Fusion

To achieve low level fusion for image set 1, the spatially registered chemical image cubes were concatenated along the wavenumber/Raman shift dimension (see Figure 2, part 1), resulting in a fused IR-Raman chemical image cube. Prior to fusion, each of the IR and Raman cubes was auto-scaled by subtracting the mean and dividing by the standard deviation of each column of the unfolded chemical image cube, followed by block and maximum scaling. Autoscaling and maximum scaling were necessary to prevent the Raman spectra from dominating in the subsequent multivariate analysis, as the range of intensities measured for the Raman data was much larger than that for the IR data, while block scaling was necessary to prevent the IR data, which had a larger number of spectral variables than the Raman data, from dominating. The classification map obtained using low level fused data of image set 1 (Figure 5) resulted in lower correctly classified pixels as compared with the prediction maps obtained using IR or Raman data alone (75.8% versus 91.1% and 93.7% respectively). However, the classification map obtained using low level fused data of image set 2 (Figure S3) resulted in higher correctly classified pixels as compared with the prediction maps obtained using NIR or Vis-NIR data alone (94.3% versus 90.9% and 93% respectively).

#### 4.2.3. Mid-Level Fusion

**Figure 6:**
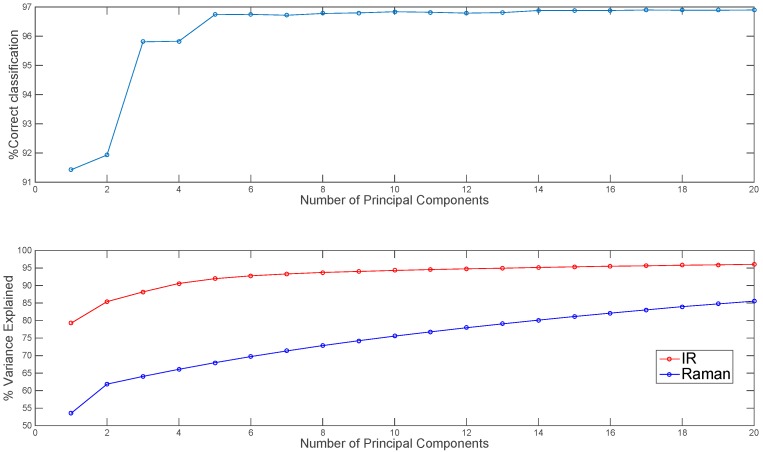
Effect of number of IR and Raman principal components used in mid-level fusion on % correct classification for image set 1 (**top** panel). Percentage variance explained by each number of PCs shown in lower panel.

Before mid-level fusion, PCA was applied separately to each chemical image cube and the resulting PC scores were concatenated. This dataset was then used for building PLS-DA classification models. The influence of the number of PC scores selected for each modality on the subsequent classification performance is shown in Figure 6. From 10–20 PCs, changes in classification performance were minimal; therefore, for image set 1, the first ten PC score images from each modality (representing 94% and 75% variance respectively) were used for mid-level fusion. These images were fused by concatenation (see Figure 2, part 2) and PLS-DA was applied to the resulting datacube. The classification map obtained using mid-level fused data (Figure 5) resulted in more correctly classified pixels as compared with the prediction maps obtained using IR or Raman data alone or low level fused data (96.8% versus 91.1%, 93.7% and 75.8% respectively). For image set 2 (see Figure S3 and Table S1), mid-level fusion also resulted in more correctly classified pixels as compared with the prediction maps obtained using NIR, Vis-NIR or low level fused data (95.1% versus 90.9%, 93% and 94.3% respectively). Mid-level fusion generally performed better than low level fusion, probably due to the inherent noise filtering effects of PCA.

#### 4.2.4. High-Level Fusion

In order to achieve high level fusion, the classification maps resulting from PLS-DA applied separately to each modality were merged by consensus—i.e., pixels classified as belonging to the same class in each class prediction map were assigned to that class in the high-level fused map, while pixels classified as belonging to different classes were classified as unknown. The consensus map suffers from the presence of several unclassified pixels, corresponding to those pixels where different classes were predicted from IR and Raman data. Consequently, for image set 1 the high level fused data resulted in the lowest classification accuracy when compared to IR only, Raman only, low and mid-level fused data (91.4% versus 91.1%, 93.7%, 75.8% and 96.8% respectively). Similar trends were observed for image set 2 (see Table S1).

#### 4.2.5. Co-Inertia Analysis

**Figure 7:**
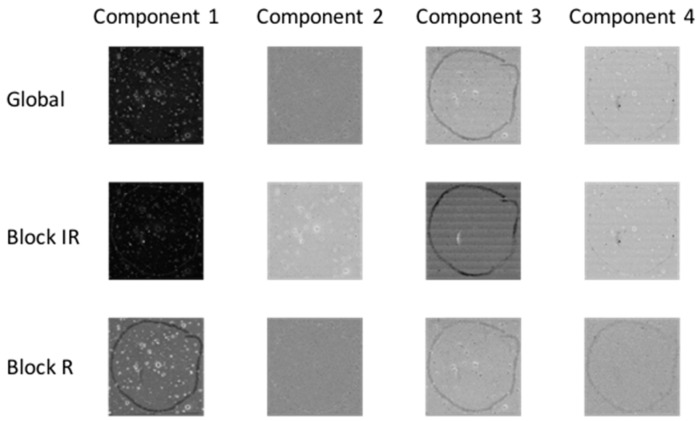
Co-inertia global and block (IR = infrared, R = Raman) score images for 1st four co-inertia components of image set 1.

Co-inertia analysis is a useful tool in chemical image fusion to evaluate the contribution of each chemical image cube (or “block”) to the common structure in the fused dataset, enabling inspection of the level of similarities within each block for a given global co-inertia component. Co-inertia metrics can be used to evaluate the portion of the covariance structure related to each block separately and the common structure from each chemical image. The global and block score images for co-inertia analysis are shown in Figure 7 (for image set 1) and Figure S4 (for image set 2). The similarity between each block and global component can be quantified by calculating the contribution of each block to the global scores and loadings, see Table 2 (for image set 1) and Table S2 (for image set 2). For example, consider the first co-inertia component of image set 1: the IR block loading contributes to 59% of the global loading, while the Raman block loading contributes 41%. The IR block score contributes 31% to the global score, while the Raman block score contributes 22%. Thus the total common contribution of the IR and Raman blocks to the global component is about 53%. The common contribution of each block to the 1st global component can also be evaluated using the correlation measurement, which is 0.9 for the IR block and 0.8 for the Raman block. This can be also seen on visual inspection of global and block scores shown in Figure 7: the global score for component 1 shares features of both the IR and Raman block scores. Conversely, the second global component is dominated by the Raman block, which can be seen by visual inspection of the scores in Figure 7 and the correlation values (0.98 for the Raman block and 0.3 for the IR block) given in Table 2. This analysis can be extended to higher order co-inertia components. Co-inertia analysis thus provides a simple yet powerful approach to investigating correlated and complementary information in a set of chemical images.

#### 4.2.6. Correlation Analysis

**Figure 8:**
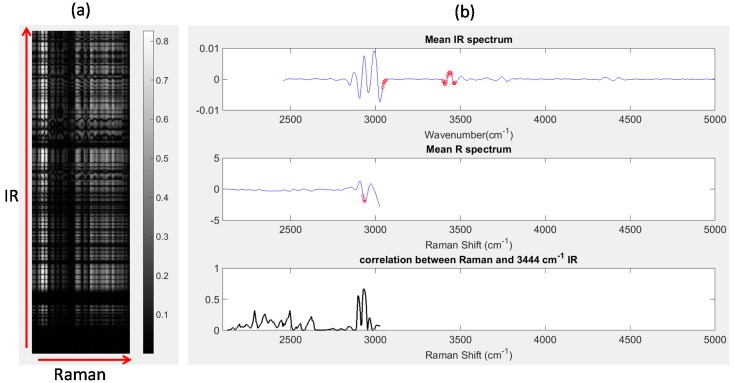
Correlation analysis: (**a**) correlation map showing correlation between all IR wavenumber and Raman shifts; (**b**) top panel: mean IR spectrum, red dots indicating IR wavenumbers with correlation coefficients > 0.8, middle panel: mean Raman (R) spectrum, red dots indicating Raman shifts with correlation coefficients > 0.8; lower panel: correlation between all Raman shifts and the IR peak at 3444 cm^−1^. This corresponds to a horizontal slice of the correlation map at the row corresponding to 3444 cm^−1^.

Image set 1: After unfolding the IR and registered Raman chemical image cubes, the Pearson correlation coefficient between each wavenumber variable in the IR chemical image cube and each Raman shift variable in the registered chemical image cube was calculated. The absolute values of the resultant correlations are displayed as a correlation map in Figure 8a, where the IR wavenumbers are represented along the y-axis, Raman wavenumber shifts are represented along the *x*-axis and bright pixels indicate regions of high correlation. Correlation maps such as those shown in Figure 8a are a simple and useful method for locating highly correlated variables across different modalities. For example, IR wavenumbers and Raman shifts with correlation coefficients greater than 0.85 are shown as red dots in Figure 8b. The IR wavenumbers with correlations greater than 0.85 were: 3047–3055 cm^−1^, 3402–3417 cm^−1^, 3425–3448 cm^−1^ and 3460–3464 cm^−1^,while the Raman shifts with correlations greater than 0.85 were: 2928–2932 cm^−1^. It is possible to inspect the correlation between any wavenumber and all Raman shifts (or vice versa), by plotting slices of the correlation map. For instance, in Figure 8b (lower panel), the absolute value of the Pearson correlation coefficient between each Raman shift and the IR peak at 3444 cm^−1^ is shown. It can be seen that the Raman shifts with highest correlations correspond to the peaks at 2928 cm^−1^ and 2932 cm^−1^. 

Image set 2: Correlation maps for image set 2 are shown in Figure S5, with regions of high correlation shown in white. The correlation between NIR wavelength 1384 nm and all Vis-NIR wavelengths is also shown in Figure S5.

### 4.3. Cross Modality Prediction

#### 4.3.1. Prediction at Low Resolution

**Figure 9:**
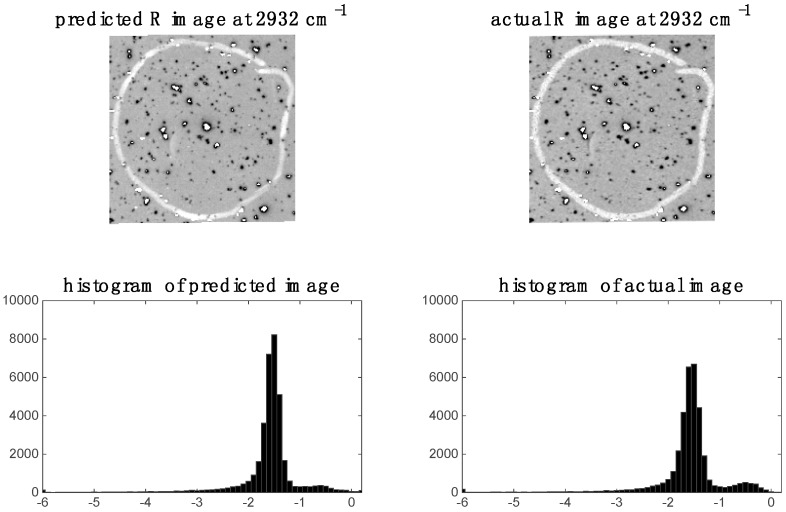
Raman image at 2932 cm^−1^: left hand side shows image (and corresponding histogram) predicted by PLS regression model applied to IR data; right hand side shows actual down-sampled Raman (R) image (and corresponding histogram) at 2932 cm^−1^.

**Figure 10:**
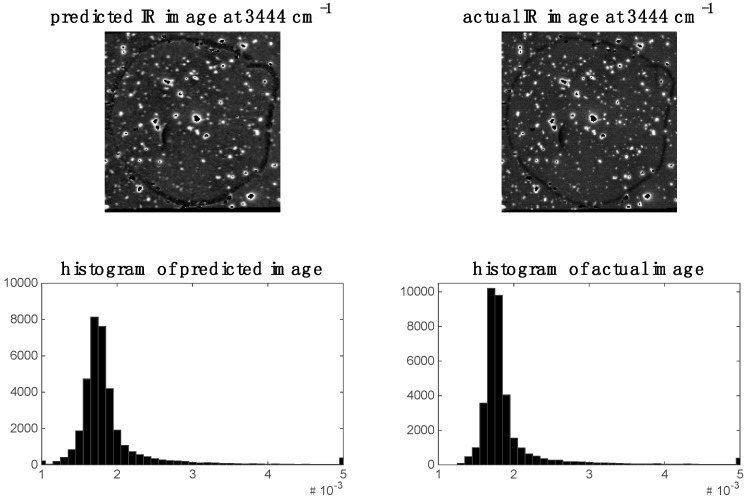
Prediction of IR image at 3444 cm^−1^ for image set 1: left hand side shows image (and corresponding histogram) predicted by PLS regression model applied to Raman data; right hand side shows actual IR image (and corresponding histogram) at 3444 cm^−1^.

In the previous section, it was shown that some spectral variables are highly correlated between modalities for image sets 1 and 2. This indicates the possibility of predicting one modality from the other. We demonstrate this by applying PLS regression to predict chemical images at specific spectral variables (i.e., wavenumbers for images set 1 and wavelengths for image set 2). For image set 1, we chose to predict the 2932 cm^−1^ Raman shift since, as shown in the previous section, this shift was highly correlated to the IR data. The predicted Raman image at 2932 cm^−1^ and its corresponding histogram are shown in Figure 9. It is evident that the predicted image corresponds well to the actual Raman image at 2932 cm^−1^. When predicting IR from Raman spectra, we chose to predict the 3444 cm^−1^ wavenumber IR image since, as shown in the previous section, this shift was highly correlated to the Raman data. The predicted Raman image at 3444 cm^−1^ and its corresponding histogram are shown in Figure 10. It is evident from these that the predicted image corresponds well to the actual IR image at 3444 cm^−1^. 

**Figure 11:**
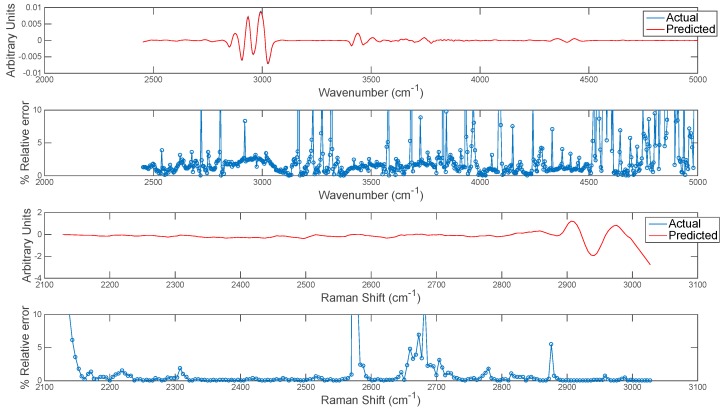
Error estimation for prediction of each wavenumber in image set 1. Top two panels show (**upper** panel) mean actual and predicted spectrum and (**lower** panel) % relative error at each wavenumber for prediction of IR from Raman chemical image data. Bottom two panels show (**upper** panel) mean actual and predicted spectrum and (**lower** panel) % relative error at each wavenumber for prediction of Raman from IR chemical image data.

The extension of this concept to predicting entire chemical image cubes from one modality to another is trivial, although care should be taken in interpreting predicted images at wavenumbers/shifts where correlation is low. In order to evaluate this, the entire chemical image cube of each modality was predicted from the other and the resultant mean spectra of the predicted and actual IR and Raman cubes are plotted in Figure 11. The relative error of prediction at each wavenumber variable was also calculated as plotted in Figure 11. The relative error could be used to define error tolerances (e.g., <2%) for prediction and to determine poorly predicted spectral variables. This information can also be used in conjunction with the correlation maps developed in the previous section: i.e., spectral variables with low correlation among modalities correspond to variables with high relative error.

Cross modality prediction images for image sets 2 and 3 are shown in the supplementary material, Figures S6, S7 and S9. The prediction maps look very similar to the actual single channel images. However, as it can be seen from the prediction maps for image set 2, noise artefacts in the calibration images can affect the prediction maps (see Figure S6). 

#### 4.3.2. Resolution Enhancement

**Figure 12:**
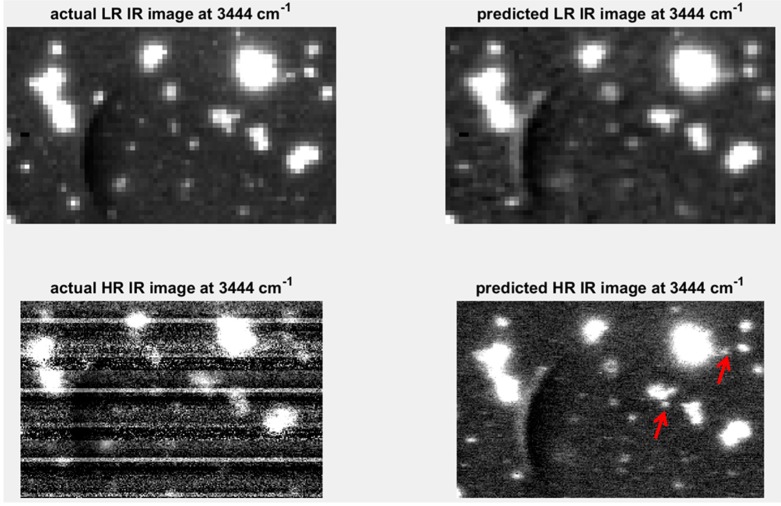
Resolution enhancement of IR image at 3444 cm^−1^: **upper left** shows low resolution (LR: pixel size = 25 µm) image; **upper right** shows low resolution IR image predicted by down-sampled Raman spectra; **lower left** shows high resolution (HR: pixel size = 6.2 µm) IR image predicted by PCA de-noised Raman spectra; **lower right** hand side shows actual HR IR image at 3444 cm^−1^. Red arrows indicate objects resolvable in the IR predicted image at 3444 cm^−1^.

A further extension of the concept of predicting one modality from another, described in the previous section, is the appealing proposition of being able to predict high spatial resolution maps using a modality with inherently higher spatial resolution [9]. Raman imaging, for example, is capable of inherently higher spatial resolution than IR imaging. Using the predictive models developed in Section 4.3.1, high spatial resolution IR chemical image cubes were predicted from the original high spatial resolution Raman chemical image cube. The predicted image was evaluated by comparison with the actual high spatial resolution IR image. Applying the predictive PLSR models directly on the original data led to the introduction of noise in the predicted high resolution image. This is because the down-sampling step in the image registration process resulted in smoothing of the Raman spectra (see Supplementary Materials Figure S11). In order to de-noise the original Raman spectra, a PCA compression step, where PCA is applied to the original datacube and the first ten PCs were used to reconstruct the cube, was carried out. The resultant PCA de-noised spectra (see Supplementary Materials Figure S11) were used as inputs to the PLSR prediction model developed on the down-sampled data, resulting in the prediction map shown in Figure 12. When compared to the actual high resolution (HR) IR image, it is clear that the level of noise in that image, due to instrumental noise related to the linear array in the IR imaging instrument, prevents the resolution of smaller objects. However, the predicted high resolution image allows the resolving of several smaller objects which were not distinguishable from each other in the low resolution images (see red arrows in Figure 12).

Similarly, high resolution prediction maps for image set 3 are shown in Figure S10. Although similar features in the resolution target are resolved in both the predicted and actual image, comparison of the image histograms is affected by noise in the predicted image (bright dots corresponding to bad pixels in the HR Raman image) and noise in the actual HR image (lines introduced by the linear array). 

## 5. Experimental Section

### 5.1. Materials and Methods

#### 5.1.1. IR Instrumentation

IR images were acquired using a Nicolet iN10 MX Imaging system by Thermo Scientific (Madison, WI, USA). The detector used was an LN2 Cooled MCTA linear array detector, which was liquid nitrogen cooled. High resolution images were obtained using the zoom feature of the system.

#### 5.1.2. Raman Instrumentation

Raman images were acquired using an InVia Micro-Raman spectroscopy system (Renishaw, Wotton-under-Edge, Gloucestershire, UK) with a 10× objective and 532 nm laser. The detector used was a NIR enhanced Deep Depletion CCD array (1024 × 256 pixels), which was Peltier cooled to −70 °C.

#### 5.1.3. NIR Instrumentation

NIR chemical images were recorded using a line-mapping NIR hyperspectral imaging system (DV Optics, Padua, Italy), working in the range 950–1650 nm, where reflectance was measured every 7 nm (further details of the instrument can be found in [1]). The pixel size obtained using this instrument was approximately 320 × 320 μm.

#### 5.1.4. Vis-NIR Instrumentation

Vis-NIR chemical images were recorded using a line-mapping hyperspectral imaging system, working in the range 450–950 nm, where reflectance was measured every 5 nm (further details of the instrument can be found in [12]). The pixel size obtained using this instrument was approximately 170 × 170 μm.

### 5.2. Image Sets

#### 5.2.1. Image Set 1

This image set consisted of IR and Raman images of a polymer blend sample of 50:50 PLA:PHB, prepared by drop casting as described in [13]. A circular marker was drawn on the centre of the sample and a spatial region around this marker was imaged using the IR microscope in transmission mode at low (25 μm) and high resolution (6.2 μm). A similar region was imaged using the Raman microscope at 10× magnification (corresponding to pixel sizes of 6.2 × 6.5 μm).

#### 5.2.2. Image Set 2

This image set consisted of NIR and Vis-NIR images of a mixture of polymer fragments (low-density polyethylene (LDPE), high-density polyethylene (HDPE), polypropylene (PP) and polystyrene (PS)). The images have two sides, the left side consisting of a piece of each polymer type and the right side consisting of a mixture of seven pieces of each polymer type. Similar regions were imaged by the NIR instrument (pixel size approximately 320 × 320 μm) and the Vis-NIR instrument (pixel size approximately 170 × 170 μm).

#### 5.2.3. Image Set 3

This image set consisted of IR and Raman images of group 5 of the 2″ × 2″ Positive, 1951 USAF Hi-Resolution Target (Edmund Optics, Ltd., Barrington, NJ, USA). The IR image was obtained in transmission mode at low (25 μm) and high resolution (6.2 μm). A similar region was imaged using the Raman microscope at 10× magnification (corresponding to pixel sizes of 6.25 × 6.25 μm).

### 5.3. Data Analysis

All data analysis was carried out using Matlab (release R2014b, The MathWorks, Inc., Natick, MA, USA) incorporating functions from the Image Processing and Statistics toolboxes and additional functions written in-house. Example data (Image set 2) and associated Matlab scripts are available on our research group’s website [14]. 

#### 5.3.1. Data Pre-Processing

Chemical image cubes were subjected to bad pixel detection prior to analysis using the method described in [15]. Spectra were subjected to second derivative Savitsky-Golay pre-treatment (window size: 15, polynomial order: 3) to reduce effects due to uneven illumination and/or sample morphology. For image set 1 spectra from each modality were auto, block and maximum-scaled prior to fusion. For image set 2 block scaling was not required as each data block had the same number of spectral variables.

#### 5.3.2. Co-Inertia Analysis

Co-inertia analysis was carried out as described in [7]. The contribution of each data block to the global scores was evaluated using the metrics described in Table 3.

### 5.4. PLS-DA Model Building

PLS-DA models were built to discriminate between the different classes in image sets 1 and 2. For each image set, the images were split into two halves: one half for model building and the other half for prediction. The first half of the image was further split into calibration and validation sets. N-PLS-DA models were built; the number of latent variables was decided on the global percentage correct classification and class membership was designated based on maximum probability. The functions for building N PLS-DA models and applying them to chemical images (Npls_da_N.m and Npls_da_N_apply_Im.m) can be found within the downloadable files in [14]. 

### 5.5. PLSR Model Building

PLSR models were developed as follows to predict IR spectra from Raman spectra and vice versa. The registered chemical image cube data was divided into calibration, training and test datasets and PLSR models were developed to predict IR or Raman chemical image intensity at a given wavenumber or Raman shift. The number of latent variables to include in each model was estimated using the method described in [16]. The functions for building PLSR models (PLS_output2.m) can be found within the downloadable files on [14]. 

## 6. Conclusions

In this paper we have presented a new framework for data fusion of chemical images, using the multivariate nature of this data to enable cross modality image registration, improved pixel level classification and improved resolution through the development of multivariate prediction models. The framework proposed is generic in that it can be applied to fuse chemical or hyperspectral data from any modality. In the example datasets studied, sufficient complementary and correlated information was available across IR and Raman modalities to demonstrate the benefits of multivariate chemical image fusion. However, some limitations of the proposed approach should be noted. Firstly, in order to perform successful multivariate chemical image registration using the proposed approach, it is a requirement that some salient features be present in the principal component score images of each modality. If two techniques are uncorrelated, it is unlikely that this requirement will be met. However, this could be overcome by the addition of landmarks, or use of edge features. Should this not be feasible, it is possible to use the approach of Allouche et al. [7] where each image is registered to a reference brightfield image. Secondly, in order for pixel classification to be improved by multivariate chemical image fusion, it is generally required that each modality contain some complementary information. The amount of complementary information between each modality can be evaluated with co-inertia analysis. An exception to this requirement would be in cases where the multivariate fusion process results in denoising of the respective modalities which may also improve classification. Finally, in order to achieve improved spatial resolution via multivariate prediction of one modality from another, it is necessary that a significant correlation exists across the modalities investigated. Adherence to the requirements stated above will generally be sample and modality dependent.

## Figures and Tables

**Table 1:** Classification performance of PLS-DA pixel classification applied to separate IR, separate Raman, low-, mid- and high-level fused IR-Raman data for image set 1 in terms of % correct class and number of latent variables (#LV) used in each model. Corresponding pixel classification maps are shown in Figure 6.

Dataset	IR only	R only	Low	Mid	High
% Correct Class	91.10%	93.70%	75.80%	96.80%	86.90%
#LV in PLS-DA	3	4	7	6	7

**Table 2:** Co-inertia analysis: contribution of block to global loadings and components and correlation between block and global scores for image set 1.

Component	1	2	3	4	5	6
Contribution of IR block loadings to global loadings (%)	58.96	25.41	46.34	79.32	76.41	3.55
Contribution of R block loadings to global loadings (%)	41.04	74.59	53.66	20.68	23.59	96.45
Contribution of IR block components to global components (%)	31.06	1.93	12.84	34.09	30.45	0.02
Contribution of R block components to global components (%)	22.28	40.1	39.14	3.79	2.01	60.67
Correlation between IR block scores and global scores	0.889	0.286	0.677	0.967	0.976	0.03
Correlation between R block scores and global scores	0.813	0.987	0.942	0.389	0.283	1

**Table 3:** Metrics derived from co-inertia analysis (u = global loading, s = global score, k = block subscript, c = component superscript, X_k_ = kth data block (i.e., unfolded chemical image)).

Contribution of block loadings to global loadings (%)	$\frac{100\times u_{k}^{\left(c\right)}}{u^{\left(c\right)}}$
Contribution of block components to global components	$\frac{100\times cov^{2}\left(s_{Xk}^{\left(c\right)}s^{\left(c\right)}\right)}{\sum_{k=1}^{K}\text{var}{\left(s_{Xk}^{\left(c\right)}\right)}^{2}}$
Correlation between IR block scores and global scores	Pearson linear correlation coefficient between block and global scores

## Supplementary Materials

Supplementary materials can be accessed at: http://www.mdpi.com/1420-3049/21/7/870/s1.
