# Supplementary material for: Multivariate Chemical Image Fusion of Vibrational Spectroscopic Imaging Modalities
Source: Molecules. 2016 Jul 2;21(7):870. doi: 10.3390/molecules21070870 (PMC6273129; doi:10.3390/molecules21070870)
Supplement: Supplementary file 1 [file molecules-21-00870-s001.pdf]

Supplementary Materials: Multivariate Chemical Image Fusion of Vibrational Spectroscopic Imaging Modalities

Aoife A. Gowen and Ronan M. Dorrepaal

1. SM1: Analysis of Image Set 2

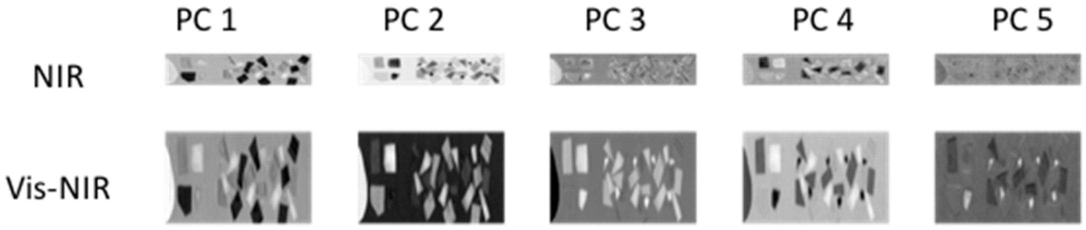

Figure S1. PCA applied to chemical image cubes from different modalities (image set 2): First five NIR and Vis-NIR PC score images are shown. The main salient features used for registration were the dark regions in PC 1. All images scaled to range mean ± 4 standard deviations.

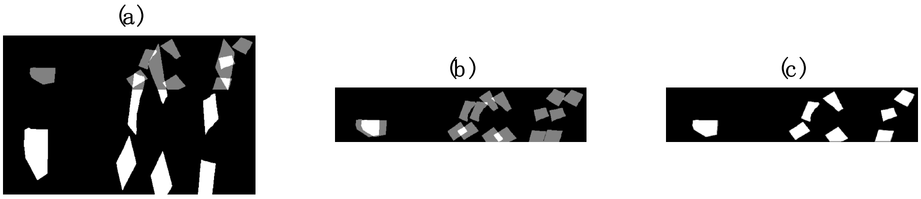

Figure S2. Multivariate image registration for image set 2. Gray pixels show mis-matching pixels, white pixels show matching pixels. (**a**) Original NIR and Vis-NIR mask images overlaid; (**b**) NIR and Vis-NIR mask images overlaid after down-sampling the Vis-NIR mask; (**c**) NIR and Vis-NIR mask images overlaid after down-sampling and applying affine transformation to the Vis-NIR mask.

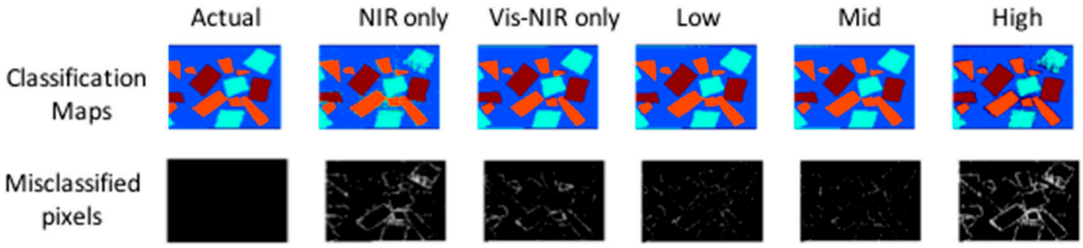

Figure S3. PLS-DA pixel classification applied to separate NIR, separate Vis-NIR, low-, mid- and high-level fused NIR-Vis-NIR data for image set 2. Actual target classification map is shown in leftmost upper panel. Classification maps for Class 1–5 resulting from PLS-DA modelling shown in upper panels. Misclassified pixels for each model shown as white pixels in lower panel. Further model details are shown in Table S1.

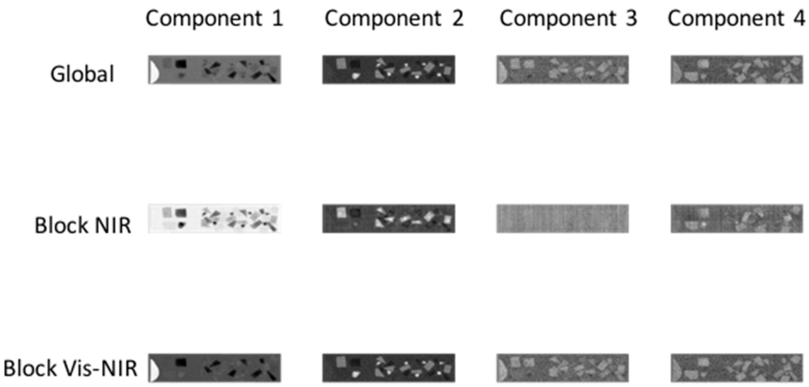

Figure S4. Co-inertia global and block (NIR = near infrared, Vis-NIR = Visible-near infrared) score images for 1st four co-inertia components of image set 2.

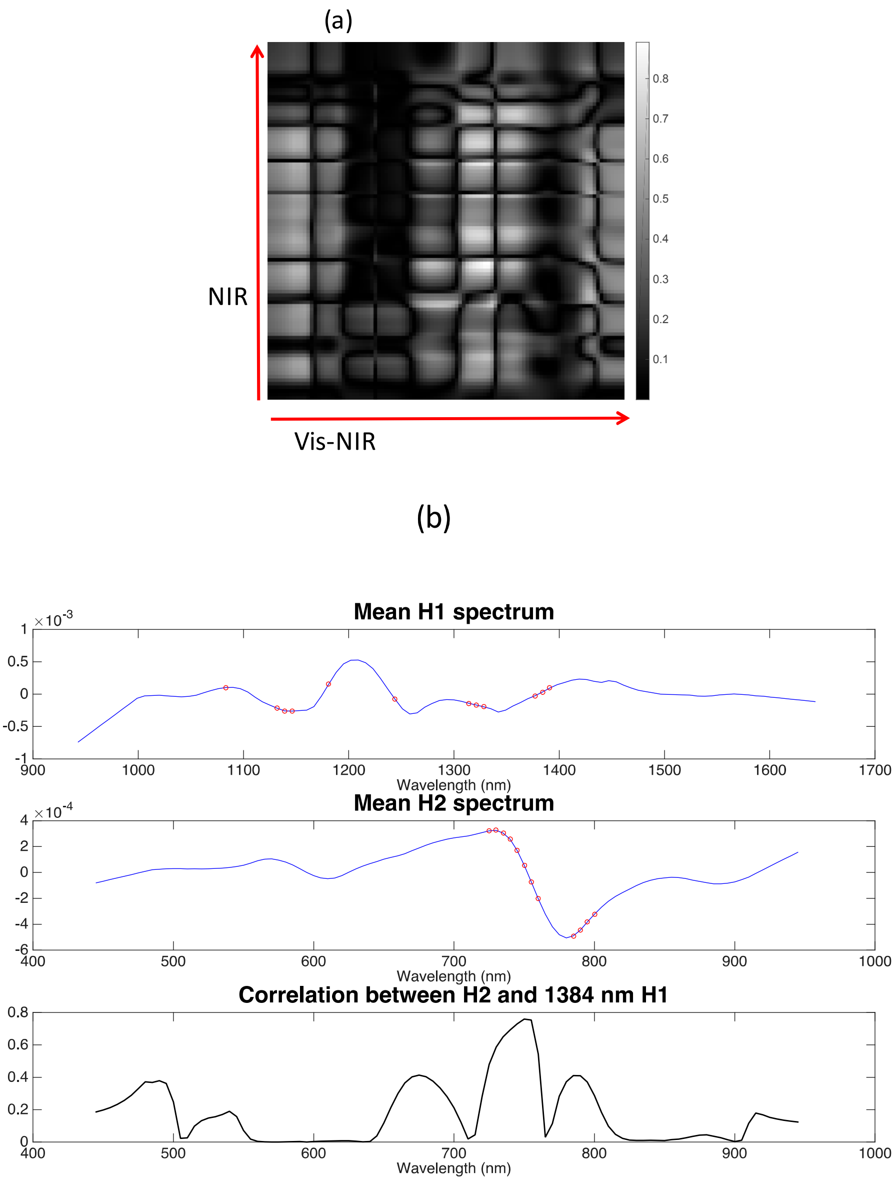

Figure S5. Correlation analysis: (**a**) correlation map showing correlation between all NIR and Vis-NIR wavelengths; (**b**) top panel: mean NIR spectrum, red dots indicating NIR wavelengths with correlation coefficients > 0.8, middle panel: mean Vis-NIR spectrum, red dots indicating Vis-NIR wavelengths with correlation coefficients > 0.8, lower panel: correlation between all Vis-NIR wavelengths and the NIR peak at 1384 nm. This corresponds to a horizontal slice of the correlation map at the row corresponding to 1384 nm.

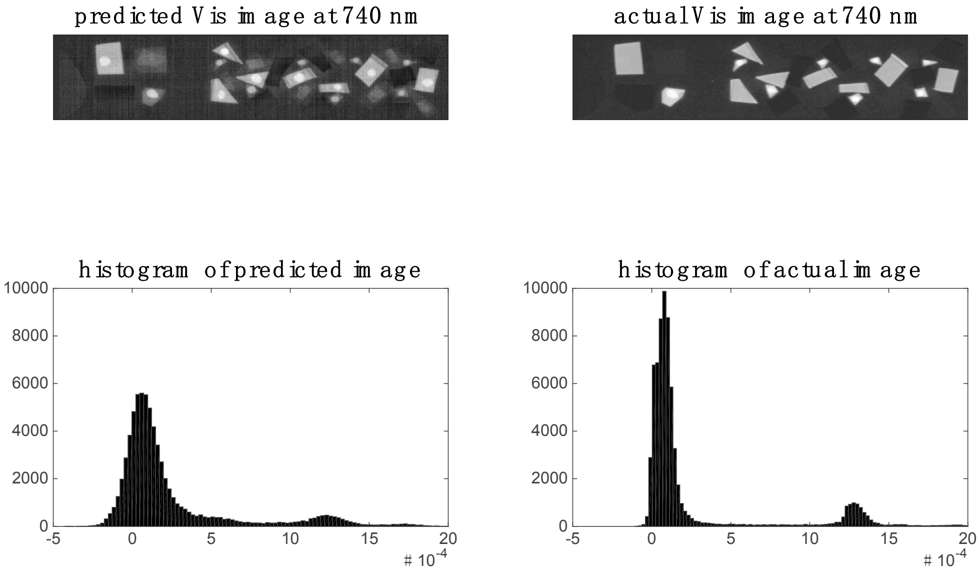

Figure S6. Vis-NIR image at 740 nm: left hand side shows image (and corresponding histogram) predicted by PLS regression model applied to NIR data; right hand side shows actual down-sampled Vis-NIR image (and corresponding histogram) at 740 nm.

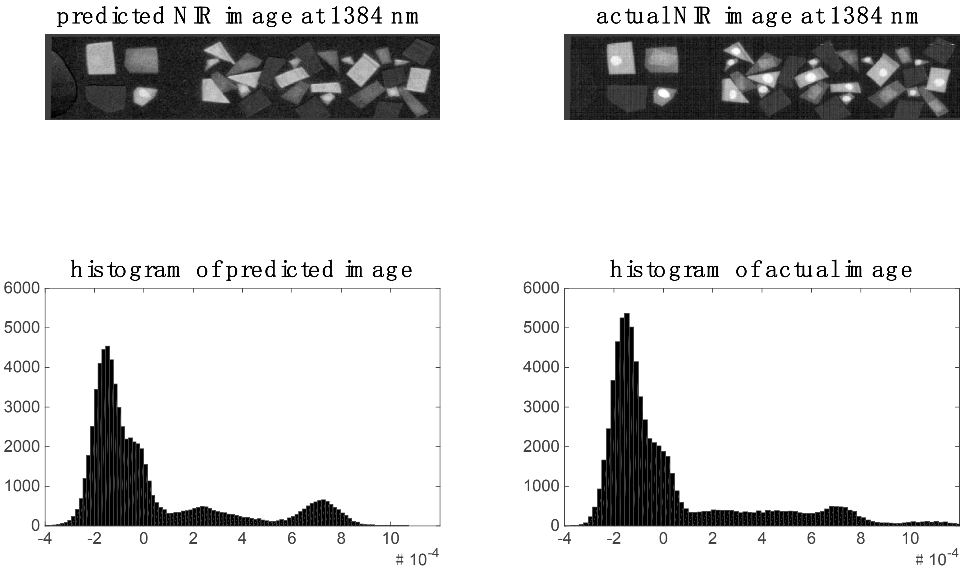

Figure S7. Prediction of NIR image at 1384 nm for image set 2: left hand side shows image (and corresponding histogram) predicted by PLS regression model applied to Vis-NIR data; right hand side shows actual NIR image (and corresponding histogram) at 1384 nm.

Table S1. Classification performance of PLS-DA pixel classification applied to separate NIR, separate Vis-NIR, low-, mid- and high-level fused NIR-Vis-NIR data in terms of % correct class and number of latent variables (#LV) used in each model. Corresponding pixel classification maps are shown in Figure S3.

Dataset	NIR only	Vis_NIR only	Low	Mid	High
% Correct Class	90.90%	93%	94.30%	95.10%	88.10%
#LV in PLS-DA	10	7	5	5	17

Table S2. Co-inertia analysis: contribution of block to global loadings and components and correlation between block and global scores for image set 2.

Component	1	2	3	4	5	6
Contribution of NIR block loadings to global loadings (%)	38.43	16.77	8.82	7.54	5.24	7.73
Contribution of Vis-NIR block loadings to global loadings (%)	61.57	83.23	91.18	92.46	94.76	92.27
Contribution of NIR block components to global components (%)	11.73	2.82	0.71	0.53	0.21	0.31
Contribution of Vis-NIR block components to global components (%)	42.66	90.11	59.06	94.94	91.88	54.43
Correlation between NIR block scores and global scores	0.66	0.675	0.198	0.438	0.226	0.125
Correlation between Vis-NIR block scores and global scores	0.954	0.999	0.998	1	1	0.999

2. SM2: Analysis of Image Set 3

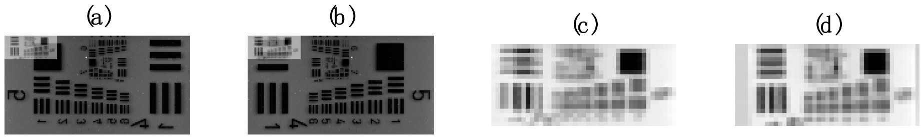

Figure S8. Multivariate image registration for image set 3. (**a**) Original IR and Raman PC images overlaid; (**b**) IR and Raman PC images overlaid after rotating and flipping Raman mask; (**c**) IR and Raman PC images overlaid after rotating, flipping and down-sampling the Raman mask; (**d**) IR and Raman PC images overlaid after rotating, flipping, down-sampling and applying affine transformation to the Raman mask.

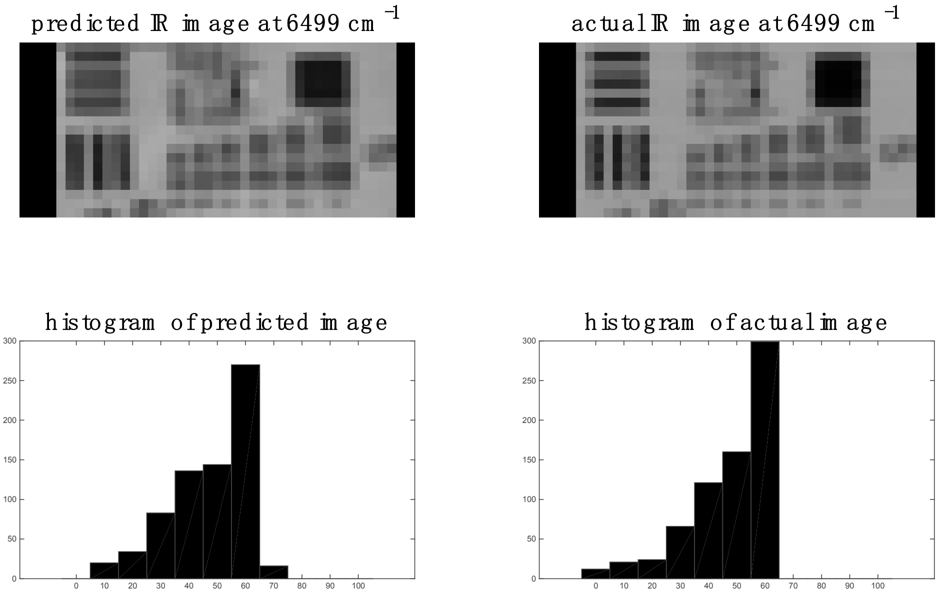

Figure S9. Prediction of IR image at 6499 cm^{-1} for image set 3: left hand side shows image (and corresponding histogram) predicted by PLS regression model applied to Raman data; right hand side shows actual IR image (and corresponding histogram) at 6499 cm^{-1}.

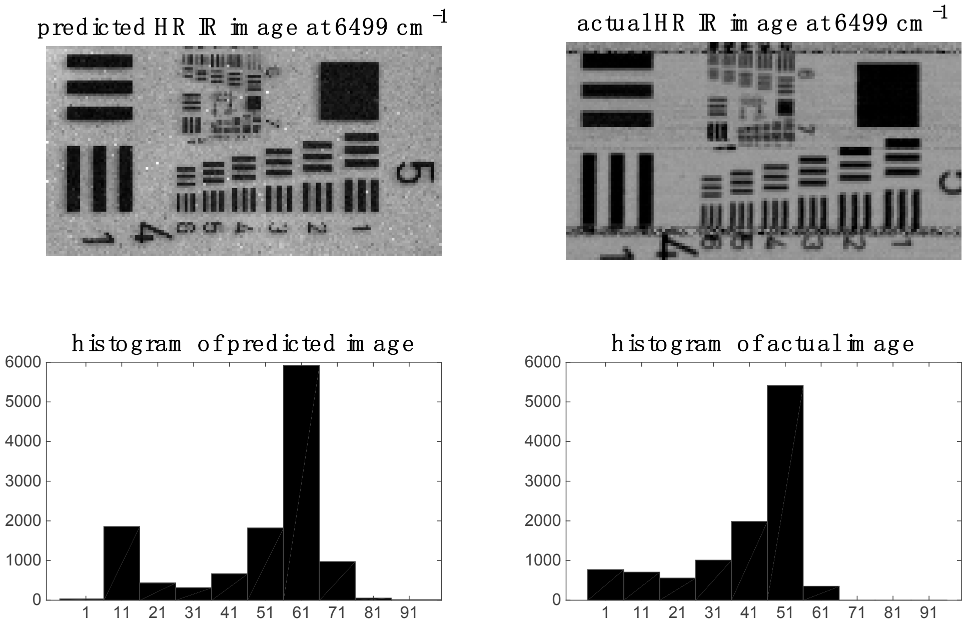

Figure S10. Resolution enhancement of image set 3, IR image at 6499 cm^{-1}: left hand side shows high resolution (HR: pixel size = 6.2 µm) IR image and histogram predicted by PCA de-noised Raman spectra; right hand side shows actual HR IR image and histogram at 6499 cm^{-1}.

3. SM3: Resolution Enhancement

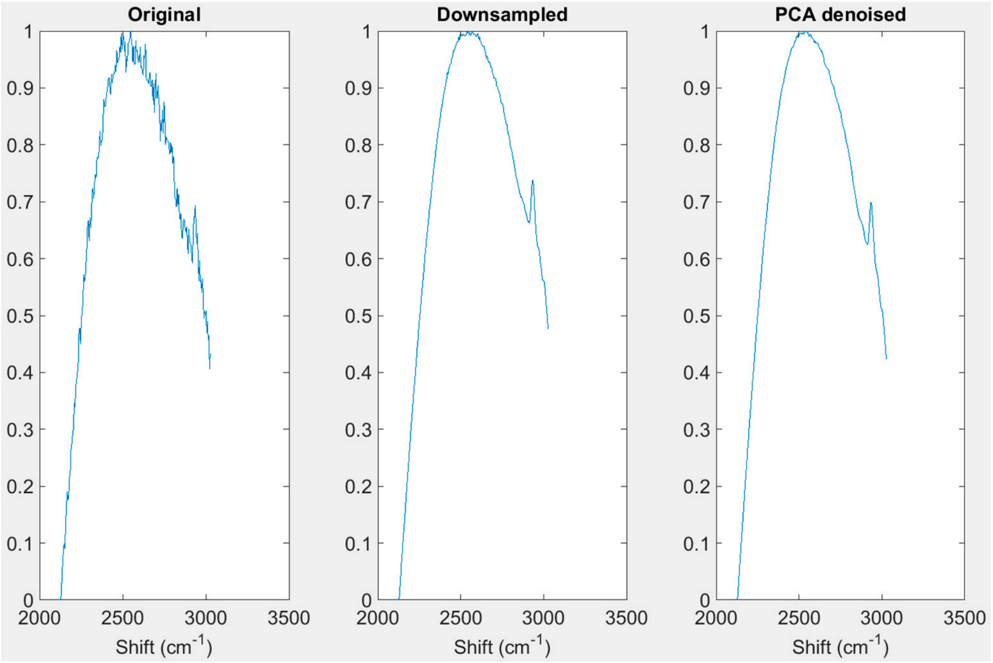

Figure S11. Effect of down-sampling and PCA de-noising on Raman pixel spectra.

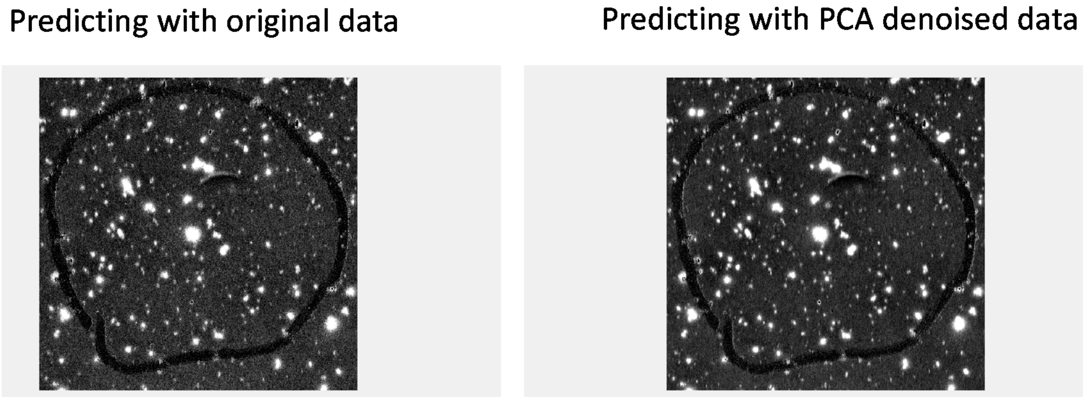

Figure S12. Effect of PCA de-noising on prediction of IR chemical images from Raman spectra.
